# Pay for performance, satisfaction and retention in longitudinal crowdsourced research

**DOI:** 10.1371/journal.pone.0245460

**Published:** 2021-01-20

**Authors:** Elena M. Auer, Tara S. Behrend, Andrew B. Collmus, Richard N. Landers, Ahleah F. Miles

**Affiliations:** 1 Department of Psychology, University of Minnesota, Minneapolis, Minnesota, United States of America; 2 Department of Organizational Sciences and Communication, George Washington University, Washington, District of Columbia, United States of America; 3 Department of Psychology, Old Dominion University, Norfolk, Virginia, United States of America; Universitat de Valencia, SPAIN

## Abstract

In the social and cognitive sciences, crowdsourcing provides up to half of all research participants. Despite this popularity, researchers typically do not conceptualize participants accurately, as gig-economy worker-participants. Applying theories of employee motivation and the psychological contract between employees and employers, we hypothesized that pay and pay raises would drive worker-participant satisfaction, performance, and retention in a longitudinal study. In an experiment hiring 359 Amazon Mechanical Turk Workers, we found that initial pay, relative increase of pay over time, and overall pay did not have substantial influence on subsequent performance. However, pay significantly predicted participants' perceived choice, justice perceptions, and attrition. Given this, we conclude that worker-participants are particularly vulnerable to exploitation, having relatively low power to negotiate pay. Results of this study suggest that researchers wishing to crowdsource research participants using MTurk might not face practical dangers such as decreased performance as a result of lower pay, but they must recognize an ethical obligation to treat Workers fairly.

## Introduction

In the social and cognitive sciences, crowdsourcing [1] provides up to half of all research participants [2] and is growing in popularity, with Amazon Mechanical Turk (MTurk) as a dominant source [3]. For researchers, crowdsourcing has provided access to a large, diverse, and convenient pool of participants. Referred to by Amazon as Workers, we conceptualize these individuals as “worker-participants” based on their self-identification as workers and their role as research participants. Past research suggests that although the characteristics of individuals participating in crowdsourcing may be somewhat idiosyncratic, these differences do not generally threaten the conclusions of studies, with some exceptions (e.g., nonnaiveté; [1,4,5]).

As platforms such as MTurk reach maturity, the dual goals of ensuring scientific validity and protecting worker-participants’ rights must both be met. Past research in this area has focused almost exclusively on questions of issues of generalizability and representativeness in relation to other samples (e.g. [4,5]) but has not generally considered the ethical and practical implications of sampling from a population of gig-economy contract workers versus more traditional populations.

MTurk Workers share common characteristics with other paid research participants and may be conceptualized as professional subjects whose participation in academic research is solely to generate income. Previous research has examined unique behaviors of professional subjects such as deception in screening questionnaires to ensure selection for participation [6]. The MTurk Worker population is comprised of both professional research subjects and individuals who engage in various other Human Intelligence Tasks (HITs) outside of academic research (e.g., editing a computer-generated transcription of an audio file). They have made efforts to self-identify as contract employees and publicize their expectations of the Worker-Requester relationship in addition to other behaviors that indicate a nontraditional work environment with characteristics of both professional subjects and gig contract workers in an employment setting.

All academic research is bound by an ethical code that is concerned with protecting the rights of human subjects, explicated in guidelines such as the Belmont Report and enforced by the review boards of academic institutions and other governing bodies [7]. However, it is important to consider the ways in which crowdsourced research conducted through online platforms has implications for both general research ethics and gig work. MTurk provides a unique population of independent contractors with aspects that differentiate it from other types of research participants and other types of employees, which can provide novel and useful information for both groups. MTurk allows Workers to explicitly sign up for a marketplace with the opportunity to complete tasks for pay at their own discretion. This exerts a certain market pressure on Workers that is analogous to traditional labor halls for union members seen in the industrial era [8,9]. This labor model has also seen a renaissance with day laborers in the agriculture and logistics industries [10]. This analogy can be applied to many online marketplaces such as Prolific, Upwork, and Fiver; however, we have chosen MTurk for this study based on its’ prevalent usage for social science research and the researchers’ direct payment of participants. Other types of online research panels obtain participants from many different sources with varying types of rewards or compensation (e.g., game tokens and points toward gift cards) that does not typically align with the labor hall model. In these cases, researchers may not have direct knowledge of type and level of participant incentives, but instead pay the crowdsourcing platform for the collection of a panel. This type of compensation has more definitive ethical violations than the nuances afforded by the labor market created on the MTurk platform. Thus, MTurk represents a unique subset of research participants that are also gig economy workers. This conceptualization necessarily benefits from previous research on work motivation, behavior and attitudes.

The work motivation literature [11] suggests that of the many relevant factors that may determine motivation, pay and expectations about pay stand out as especially relevant. Locke, Feren, McCaleb, Shaw, and Denny [12] argued, “No other incentive or motivational technique comes even close to money with respect to its instrumental value” (p. 379). Thus, we seek to understand the effect of pay on crowdsourced worker-participant behavior. We focused on the special case of a longitudinal study in which participants must return on a separate occasion to explore how pay affects worker-participant performance (i.e., data quality), satisfaction, and attrition.

Since the creation of MTurk in 2007, researchers have explored how pay influences behavior in crowdsourced work marketplaces. In an early study, Buhrmester et al. [3] found that relatively higher pay rates (50 cents vs 5 cents) resulted in faster overall data collection time, with no major differences in data quality as assessed by scale reliability. Litman, Robinson, and Rosenzweig [13] found that monetary compensation was the highest-rated motivation for completing a research study among US-based Workers, contrary to findings just four years prior [3]. A recent poll found that on average MTurk Workers estimated fair payment hovered just above the United States minimum wage ($7.25/hr), up from the previous standard of $6/hr [14]. The MTurk community is evolving over time, and the norms and expectations for pay have changed, with new tools constantly emerging to meet Workers’ demands for fair pay (e.g., Turkopticon, TurkerView).

As Workers develop more employee-like identities, we argue that they follow patterns explicated in pay-for-performance theory [15]. Pay predicts a number of goal-directed behaviors because it supports physiological and safety needs [16]. Classic studies have found that pay for performance leads employees to increased productivity [12,17]. Aligned with this evidence on the extrinsic motivation provided by pay, we hypothesize:

Hypothesis 1: Base pay, pay increases, and total pay positively affect the performance of worker-participants as measured by indicators of data quality.

Although pay is often critical to work motivation, meta-analytic findings suggest that in traditional forms of work, pay is only slightly related to job satisfaction [18] and performance [19]. In short, pay might encourage worker-participants to exert just enough effort to be compensated and no more. Compensation is better considered a multifaceted issue in which the level of compensation matters, but so too do worker-participants’ expectations and their understanding of their compensation. In most organizations, many aspects of the employment relationship are left unstated, yet form a *psychological contract* between the employee and employer [20–22]. Each party, the employee and employer, holds beliefs about what they expect from the other and what they are obligated to provide in return [22]. Contractual beliefs come in part from schema, norms, and past experiences [21]. When the employer and employee hold mutual beliefs, effective performance, feelings of trust and commitment, and reciprocity follow [22]. This contract is an important framework within which to understand compensation.

The development of psychological contracts also has major implications for perceived organizational justice. Specifically, distributive justice [23] which focuses on the perceptions of decision outcomes in an organization or group, has been previously applied to compensation fairness [24,25]. Typically, distributive justice is cultivated when these outcomes are aligned with norms for the allocation of rewards (i.e., equity and fair pay for good performance) [26]. Especially relevant for research participants, this concept also has roots in research ethics (see the Belmont Report; [7]).

As the norms of MTurk evolve, the expectations and the psychological contract between Workers and Requesters (i.e., those providing tasks to complete) also change. In early days, Workers did not have strong prior experience to draw from in forming expectations. Now, as a mature system, Workers have strong beliefs and have formed expectations of their employers. By examining websites such as Turkopticon, where MTurk users report violations of their self-formed Bill of Rights, it becomes clear that Workers are not traditional paid participants [27]. Among these expectations are fair pay equivalent to US minimum wage, swift payment for good work, and bonuses for outstanding work [28]. To address these motivational aspects of crowdsourced research, we hypothesize:

Hypothesis 2: Base pay, pay raises, and total pay positively affect worker-participant satisfaction as measured by intrinsic motivation, compensation reactions, and distributive justice.

As in any other workplace, trust and credibility are essential in determining whether a worker-participant will return to complete additional work. Attrition in traditional employment settings can often be attributed to dissatisfaction, depending on an employee’s job embeddedness, agency, or commitment [29–31]. Absolute pay level is also related to turnover [32], although pay raises have been demonstrated to be more important in determining both turnover and fairness perceptions [19]. The crowdsourcing environment is unusual compared to other forms of work, however, in that worker-participants have less obvious opportunities to interact with each other, reducing their likelihood of forming bonds that drive retention decisions unless they seek out online communities built for that purpose. Further, the physical environment is not fixed, removing concerns such as location and community in determining retention. Thus, in the specific context of longitudinal crowdsourced work, we hypothesize:

Hypothesis 3: Base pay, pay increases, and total pay negatively affect attrition of worker-participants.

## Method

This study was approved and monitored by the Institutional Review Board of Old Dominion University (Reference number: 15–183).

### Participants

Participants (N = 359) were adult users of MTurk located in the United States. One participant completed more than one condition and was removed from the data set. 50% of participants identified as female, 70% identified as White, 6% as African American, 14% as Asian American, 1.7% as Native American or Native Alaskan and 9.2% identified as “other.” 63% of participants reported working full-time, 16% reported working part-time and 21% were unemployed. Of those employed, about 20% were employed in business service, 12% in education, 12% in finance, 8% in healthcare, 10% in manufacturing, and 10% in retail. There were minimal differences in demographics between the initial sample and the retained sample in the second wave of the study (Table 1).

**Table 1:** Demographics by retained status.

	*Total Sample (T1)*		*Retained Sample (T2)*	
Gender				
Male	50%	179	48%	72
Female	50%	180	52%	79
Race				
Black	6%	21	7%	11
Asian	14%	49	19%	28
White	70%	250	61%	92
Other	9%	32	11%	17
Two or More	2%	7	2%	3
Employment Status				
Full time	63%	226	70%	106
Part time	16%	57	15%	22
Not employed	21%	76	15%	23
Master Worker Status				
Yes	13%	48	13%	20
No	87%	311	87%	131
Total N		359		151

*p < .05. **p < .01. ***p < .001.

### Design

We used a 3x3 between-subjects design in which the manipulated factors were Time 1 (T1) Pay (X1: $.50, $1, $2) and Pay Multiplier (X2: 100%, 200%, 400%) which represented a relative pay increase at Time 2 (T2). Thus, worker-participants who completed both waves of the study were paid anywhere between $1 and $10 total, and this total pay represents the interaction between X1 and X2. See Table 2 for a closer examination of each cell of the experimental design in addition to the observed hourly wage based on average completion time in each condition. To control for time of day effects and potential time-zone availability differences, each condition was split into two halves, which were deployed at either 12:00 p.m. or 8:00 p.m. EST. The T2 follow up for each wave was matched to the T1 day and time. The groups were made available sequentially, every 3–4 days, from January 11 to May 13. The T1 waves were each open for 12 hours, and the T2 waves were open for up to 6 weeks. The T2 deployments were accompanied by a reminder email.

**Table 2:** N, Pay, average completion time, and observed hourly wage by experimental condition.

	*T1 N*	*T2 N*	*Retention Rate*	*T1 Pay*	*T2 Pay*	*Total Pay*	*T1 Average Completion Time*^*a*^	*T2 Average Completion Time*^*a*^	*Observed Hourly Wage*^*b*^
	40	7	0.18	$0.50	$0.50	$1.00	28.66	25.47	$1.11
	40	10	0.25	$0.50	$1.00	$1.50	26.74	24.52	$1.76
	40	20	0.50	$0.50	$2.00	$2.50	33.09	23.06	$2.67
	40	14	0.35	$1.00	$1.00	$2.00	23.66	26.09	$2.41
	39	15	0.38	$1.00	$2.00	$3.00	34.73	35.49	$2.56
	40	22	0.55	$1.00	$4.00	$5.00	30.38	24.69	$5.45
	40	18	0.45	$2.00	$2.00	$4.00	38.52	37.75	$3.15
	40	17	0.43	$2.00	$4.00	$6.00	34.18	28.40	$5.75
	40	28	0.70	$2.00	$8.00	$10.00	30.73	27.72	$10.27
Total	359	151	0.42						

Note

^a^in minutes

^b^Observed Hourly Wage = (Total Pay/(T1 Average Completion Time + T2 Average Completion Time))*60.

### Procedure

Participants first viewed a recruitment notice for the task on Amazon’s MTurk and self-selected to participate. The recruitment notice included the time-to-completion estimate (30 minutes), compensation for both the current task and the follow-up, and information about the second questionnaire invitation to follow in approximately 30 days. This information was also repeated in the consent script upon acceptance of the Human Intelligence Task (HIT) on the MTurk platform. Each condition received HIT recruitment notices and consent scripts specific to their experimental manipulation. Participants gave informed consent by clicking “YES” on a consent script before proceeding to the experiment. Participants who accepted the terms were directed to complete the questionnaire containing all measures. They had 12 hours to complete the survey and were told that their choice to participate in the second part of the study would not affect their payment for part one. The last page of the questionnaire contained a unique ID to submit for payment. Six weeks later, participants were emailed with a direct invitation to participate at Time 2. Participant contact was managed within the MTurk platform. Minimal identifiable information was collected (demographics and MTurk ID for payment), and no attempts were made to re-identify individuals based on their unique MTurk ID. If the participant accepted the invitation to the second wave, they were directed to an identical survey and followed an initial set of procedures. After the study was completed for all participants, debriefing documentation was emailed to all participants.

### Measures

As the core “work”, worker-participants completed a HIT (Human Intelligence Task; a piece of work offered on the MTurk platform) comprised of a series of well-validated cognitive and personality instruments. These included a ten-item Big 5 personality measure [33], a positive and negative affectivity questionnaire [34], a 30-item cognitive ability test [35], a personal altruism questionnaire [36], the Neutral Objects Satisfaction Questionnaire (NOSQ; [37]), and an Adult Decision-making competency questionnaire [38]. Post-work attitude measures included compensation reactions, intrinsic motivation [39], and distributive justice [26]. The above measures served both as a combination of outcome variables in their own right, and a means to assess data quality and reliability.

Performance was operationalized in several ways. The first indicator of performance was the number of attention check items answered correctly at each wave. Both instructed items and bogus items were used [40]. For example, participants were asked to “Select the option that is at the left end of the scale for this question.” (see [41]). Ostensibly, anyone answering questions arbitrarily would miss some of these items. Each wave contained five attention checks. Second, personal reliability (test-retest) for two scales, personality and cognitive ability, was calculated, such that higher reliability indicated better performance [3]. Third, following recommendations from Meade & Craig [41] maximum and average LongString values were calculated representing the maximum and average number of identical responses in a row, respectively. LongString values were calculated using all non-outcome scales that would permit long-string responses including the big five personality, affect, personal altruism, and neutral objects questionnaires.

Motivation was measured using three subscales from the Intrinsic Motivation Inventory [39], including the Interest/Enjoyment Subscale (e.g., “I enjoyed doing this HIT very much”; α = .79) along with Perceived Effort/Importance (e.g., “I put a lot of effort into this.”; α = .81), and Perceived Choice (“I believe I had some choice about doing this HIT.”; α = .82).

Satisfaction with compensation was measured with two separate items regarding the current task (e.g., “I am satisfied with the overall pay I will receive for this HIT”) and overall compensation for both tasks (e.g., “I am satisfied with the overall pay I will receive for these two HITs.”) and four items from Colquitt’s [26] distributive justice scale (e.g., “Does your compensation reflect the effort you have put into your work?”; α = .86).

Retention was operationalized as successful completion of the second wave of the study.

Manipulation checks of the experimental conditions occurred in both waves of the study. Participants were asked to confirm how much money they were paid for each wave in addition to stating whether they knew this was the first or second wave of a two-part study.

## Results

Descriptive statistics and intercorrelations for all variables are in Table 3. For this study, manipulation checks served their typical purpose of flagging insufficient effort responding; this also served as one test of the effect of experimental conditions on performance [40]. Approximately 90% of participants correctly reported how much money they were paid for the first wave, 94% indicated that they were aware they were taking the first part of a two-part study, and 96% indicated that they intended to complete the second part of the study. Seventy-seven percent of participants correctly identified how much they were paid in wave two and 93% indicated that they were aware they were taking the second part of a two-part study. Although, there is not a clear pattern explaining the noticeable drop in correct pay identification in the second wave, there are a number of possible explanations including careless responding and confusion about total pay as opposed to current wave pay. Additionally, Workers often sort HITs based on pay and once they have reached a personal threshold, may not remember the exact pay for each HIT they accept. Based on the research questions being addressed in our study, retaining those individuals who did not pass the manipulation checks for the final analysis made our sample more representative of the typical MTurk population.

**Table 3:** Means, standard deviations, and bivariate correlations for study variables.

	*M*	*SD*	1	2	3	4	5	6	7	8	9	10	11	12	13	14	15	16	17	18	19	20	21	22	23	24	25	26	27	28
1. T1 Pay: $0.50	--	--																												
2. T1 Pay: $1	--	--	-.50***																											
3. T1 Pay: $2	--	--	-.50***	-.50***																										
4. Pay Increase: 100%	--	--	.00	.00	.00																									
5. Pay Increase: 200%	--	--	.00	-.01	.00	-.50***																								
6. Pay Increase: 400%	--	--	.00	.00	.00	-.50***	-.50***																							
7. T1 Attention Check	4.47	1.10	-.08	-.08*	.16**	-.04	.10	-.06																						
8. T2 Attention Check	4.44	1.18	-.07	-.20**	.25**	-.06	-.08	.12	.82***																					
9. Personality Reliability	.71	.33	-.12	-.23	.32***	-.08	.04	.04	.66***	.64***																				
10. Cognitive Ability Reliability	.50	.33	-.16	-.11	.24**	-.10	.07	.03	.23**	.23**	.31***																			
11. T1 Average LongString	4.86	2.02	.01	.03	-.04	-.06	.02	.04	.17***	.13	.25**	-.23**																		
12. T2 Average LongString	5.10	2.33	.02	.07	-.08	-.02	.11	-.08	.26***	.15	.27**	-.14	.78***																	
13. T1 Max LongString	7.98	4.45	-.05	.04	.01	-.03	-.01	.04	.17**	.12	.21**	-.19*	.92***	.65***																
14. T2 Max LongString	8.47	4.75	.03	.06	-.08	-.06	.11	-.05	.23**	.14	.29***	-.16*	.72***	.93***	.62***															
15. T1 Compensation Reaction Current HIT	3.64	1.19	-.16**	.06	.11*	-.07	.02	.05	-.16**	-.20*	-.17	-.22**	.05	.01	.05	.07														
16. T1 Compensation Reaction Both HITs	3.87	1.12	-.20***	.05	.15**	-.23***	.03	.20***	-.08	-.06	.00	-.04	-.02	-.07	.00	.02	.77***													
17. T2 Compensation Reaction Current HIT	4.13	1.03	-.19*	-.06	.22**	-.32***	.07	.22**	.00	.03	.06	.05	-.12	-.13	-.05	-.02	.38***	.58***												
18. T2 Compensation Reaction Both HITs	4.05	1.07	-.21**	-.04	.23**	-.31***	.06	.22**	-.01	-.01	.04	.03	-.06	-.11	.03	.00	.35***	.61***	.90***											
19. T1 Distributive Justice	3.51	1.12	-.16**	.04	.12*	-.07	.02	.05	-.13*	-.09	-.07	-.07	.07	-.06	.08	-.01	.68***	.60***	.32***	.35***										
20. T2 Distributive Justice	3.98	1.01	-.15	-.07	.20*	-.23**	.08	.13	.04	.07	.13	.06	.04	.00	.08	.09	.24**	.43***	.70***	.64***	.39***									
21. T1 IMI: Enjoyment	4.86	1.45	.03	.02	-.05	-.08	.05	.04	-.05	-.08	-.01	-.02	.20***	.16*	.16**	.20*	.44***	.44***	.32***	.34***	.39***	.24**								
22. T2 IMI: Enjoyment	5.05	1.49	.09	.07	-.14	-.18*	.17*	.00	-.03	.00	.06	-.03	.19*	.12	.17***	.20*	.37***	.46***	.38***	.42***	.40***	.34***	.83***							
23. T1 IMI: Effort	5.95	.99	-.07	-.01	.08	-.03	.02	.01	.48***	.39***	.48***	.15	.23***	.31***	.19***	.32***	-.04	.08	.09	.07	.01	.17*	.30***	.28***						
24. T2 IMI: Effort	5.91	1.00	-.02	-.14	.16	-.15	-.01	.13	.52***	.52***	.58***	.15	.19*	.18*	.13	.16	-.22**	-.02	.10	.14	-.05	.19*	.22**	.28***	.65***					
25. T1 IMI: Perceived Choice	5.85	1.19	-.17**	.00	.17**	-.15**	.11*	.04	.53***	.48***	.60***	.29***	.15**	.25**	.16**	.24**	.03	.13**	.16*	.20*	-.06	.15	.13*	.02	.48***	.44***				
26. T2 IMI: Perceived Choice	5.66	1.39	-.10	-.22**	.29***	-.11	.00	.09	.59***	.55***	.61***	.26**	.10	.10	.13	.10	-.07	.08	.25**	.24**	-.03	.22**	.10	.08	.42***	.52***	.75***			
27. Retained	.42	.49	-.16**	.01	.15**	-.14**	-.10	.23***	-.06	--	--	--	.11*	--	.08	--	.04	.08	--	--	.05	--	.10	--	-.04	--	-.05			
28. T1 Data Collection Time	.29	.08	-.04	.03	.01	.03	.01	-.04	.09	-.02	-.14	-.01	.02	.14	.01	.05	-.08	-.05	-.17*	-.17*	-.05	-.12	-.01	-.09	.02	-.09	.00	-.07	-.04	
29. T2 Data Collection Time	3.08	6.36	.11	-.05	-.05	-.09	.12	-.03	.00	-.01	.03	.12	-.07	-.03	-.06	.01	-.12	-.05	-.14	-.17*	-.08	-.13	-.12	-.08	.07	-.06	.03	-.06	--	.08

*p < .05.

**p < .01.

***p < .001.

All hypotheses were tested using regression with appropriate considerations for dispersion (e.g., linear, Poisson, and logistic regression). Independent variables (including the interaction term) were dummy-coded. As in any multiple regression, coefficients should be interpreted as the effect conditional on all other variables in the model being held at zero. Given the dummy coding, holding variables at zero results in an estimated effect compared to the referent group where T1 Pay = $.50 and Pay Multiplier = 100% (see table notes and [42] for more details on interpreting dummy-variable regression models). These coefficients are not an exact replication of an ANOVA framework. For this reason, regression results are presented in addition to analysis of variance or analysis of deviance tables where appropriate.

Analyses are presented for both Time 1 and Time 2 outcome measures of performance and satisfaction. As was expected and used to test H3, only a portion of participants were retained at Time 2. Given this, some cell sizes at Time 2 across conditions are quite low, possibly resulting in reduced power to detect significant effects. Differences in significant effects between Time 1 and Time 2 may not be exclusively attributed to study variables and should be interpreted with the influence of this fact in mind.

**Fig 1:**
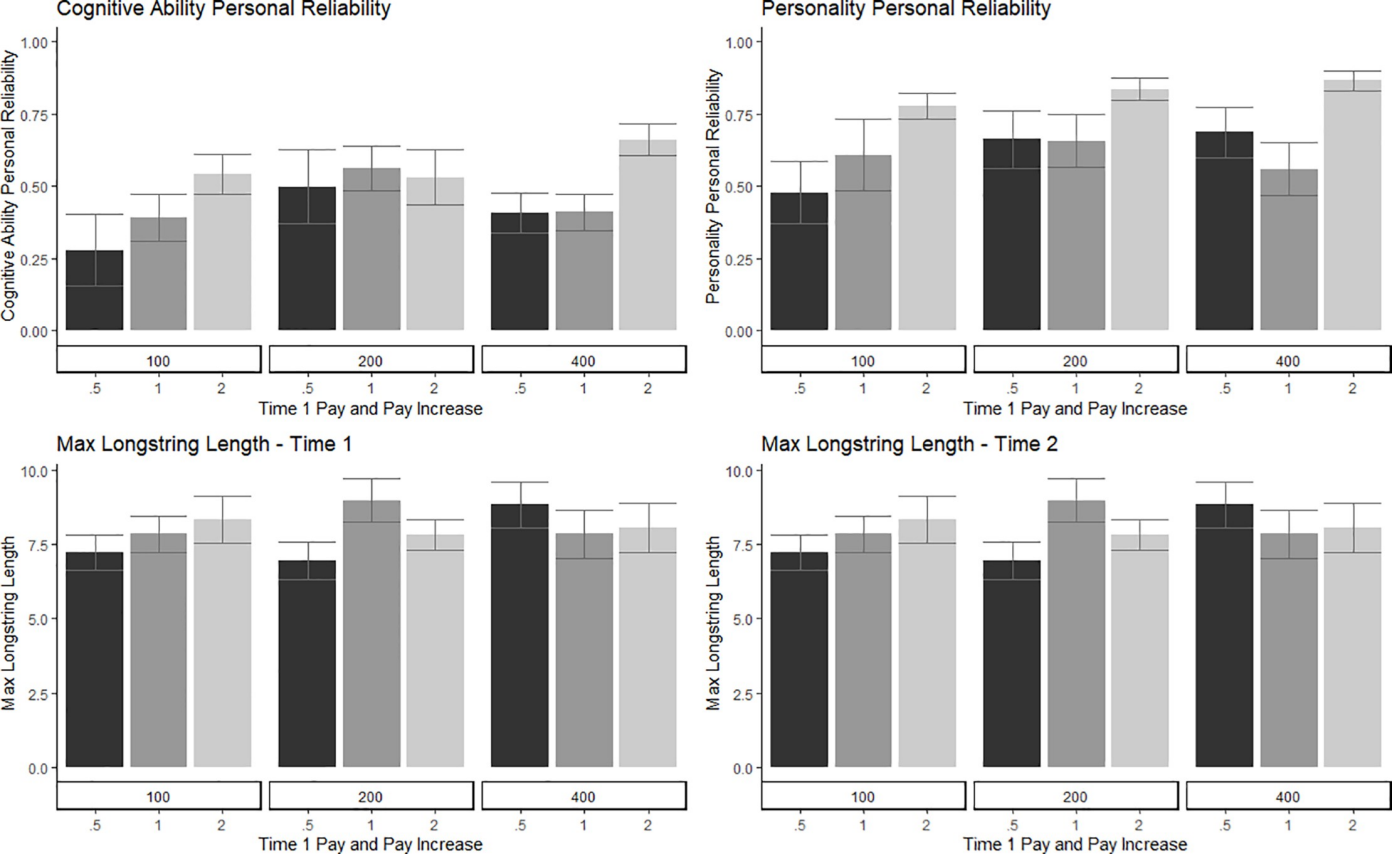
Data quality indicators by experimental pay condition.

H1 predicted that pay would positively affect worker-participant performance. The effect of pay on passed attention checks was tested using two modeling approaches, one cross-sectional and the other longitudinal. In the first model, using Poisson regression, number of passed Attention Checks at T1 was regressed on to T1 Pay, Pay Multiplier, and the interaction (Total Pay). Neither T1 Pay or Pay Multiplier significantly predicted the number of Passed Attention Checks at T1. In the second model, only including participants who completed both waves, Attention Checks passed at T2 was regressed on to T1 Pay, Pay Multiplier, and the interaction (Total Pay; Tables 4 and 5). There were no significant effects of pay on performance in the second wave of the study. The effect of pay on personal reliability, using both personality and general mental ability responses, was tested by regressing Personal Reliability scores on T1 Pay, Pay Multiplier, and the interaction (Total Pay; Tables 4 and 6, Fig 1). There was a significant effect of T1 Pay on Personality Personal Reliability scores, but not General Mental Ability. Generally, as T1 Pay increased, Personality Personal Reliability scores increased. Lastly, the effect of pay on Maximum and Average LongString values was tested (Tables 4–6, Fig 1). Total Pay had a significant effect on Maximum LongString at T1, although there was no interpretable pattern based on condition. Pay did not have a significant effect on Average LongString at T1 or T2 and did not have a significant effect on Maximum LongString at T2. To summarize, across the indicators of performance, there was no convincing evidence that initial pay or pay multiplier significantly affected data quality; H1 was not supported.

**Table 4:** Regression results for the effect of pay on worker-participant performance.

	Passed Attention Checks^a^		Personal Reliability: Personality	Personal Reliability: Cognitive Ability	Insufficient Effort—Average LongString		Insufficient Effort—Maximum Count LongString^a^	
	T1	T2			T1	T2	T1	T2
Coefficient	*B (SE)*	*B (SE)*	*B (SE)*	*B (SE)*	*B (SE)*	*B (SE)*	*B (SE)*	*B (SE)*
Intercept	1.45 (.08)***	1.42 (.19)***	0.48 (.12)***	0.28 (.12)*	4.66 (.32)***	4.86 (.89)***	1.98 (.06)***	2.13 (.13)***
Pay T1 ($1)^b^	-.02 (.11)	-.07 (.23)	0.13 (.15)	0.11 (.15)	.04 (.45)	.57 (1.09)	.08 (.08)	-.01 (.16)
Pay T1 ($2)^b^	.13 (.11)	.14 (.21)	0.3 (.14)*	0.26 (.14)	.08 (.45)	-.04 (1.04)	-.04 (.08)	-.10 (.16)
Pay Increase (200%)^c^	.09 (.11)	-.01 (.24)	0.18 (.16)	0.22 (.16)	-.04 (.45)	.09 (1.16)	.20 (.08)	.04 (.17)
Pay Increase (400%)^c^	-.02 (.11)	0.07 (.21)	0.21 (.14)	0.13 (.14)	.76 (.45)	.53 (1.03)	.17 (.11)*	.04 (.15)
Pay T1 ($1) X Pay Increase (200%)	.01 (.15)	0.06 (.31)	-0.14 (.20)	-0.05 (.20)	.66 (.64)	.28 (1.45)	-.02 (.12)	.09 (.21)
Pay T1 ($2) X Pay Increase (200%)	-.12 (.15)	-0.03 (.29)	-0.12 (.19)	-0.22 (.19)	.20 (.64)	.66(1.41)	-.02(.11)	.18 (.20)
Pay T1 ($1) X Pay Increase (400%)	.07 (.15)	0.04 (.27)	-0.26 (.18)	-0.11 (.18)	-.64 (.64)	-1.03 (1.31)	-.20 (.11)	.00 (.19)
Pay T1 ($2) X Pay Increase (400%)	-.01 (.15)	-0.04 (.25)	-0.12 (.17)	-0.01 (.17)	-.84 (.64)	-.84 (1.25)	-.23 (.11)*	-.06 (.18)
Model R^2^	.003^d^	.007^d^	.130*	.100*	-.019	.033	.008^d^	.010^d^

Note: SE = Standard error

^a^ results from Poisson regression

^b^ Time 1 Pay treated as factor for this analysis; coefficients represent the effect in comparison to the referent group of (T1 pay = $.50).

^c^ Pay increase treated as factor for this analysis; coefficients represent the effect in comparison to the referent group of (T2 increase = 100%).

^d^ McFadden R^2^ reported. Time 1 n = 359. Time 2 n = 151.

*p < .05.

**p < .01.

***p < .001.

**Table 5:** Analysis of deviance for poisson models of effect of pay on performance.

	Passed Attention Checks										Insufficient Effort—Maximum Count LongString									
	*T1*					*T2*					*T1*					*T2*				
	Residual Df	Residual Deviance	Df	Deviance	χ^2^ *p* value	Residual Df	Residual Deviance	Df	Deviance	χ^2^ *p* value	Residual Df	Residual Deviance	Df	Deviance	χ^2^ *p* value	Residual Df	Residual Deviance	Df	Deviance	χ^2^ *p* value
Null	358	141.82				150	72.25				358	754.23				150	341.49			
Pay T1	356	139.40	2	2.42	0.299	148	69.13	2	3.12	0.210	356	751.78	2	2.48	0.294	148	338.97	2	2.51	0.284
Pay Increase	354	138.39	2	1.01	0.603	146	68.37	2	0.76	0.682	354	750.20	2	1.58	0.453	146	334.02	2	4.95	0.084
Pay T1 x Pay Increase	350	137.19	4	1.20	0.879	142	68.18	4	0.19	0.996	350	736.71	4	13.49	0.009**	142	331.82	4	2.21	0.698

*p < .05.

**p < .01.

***p < .001.

**Table 6:** Analysis of variance for effect of pay on performance.

	Personal Reliability								Insufficient Effort—Average LongString							
	*Personality*				*Cognitive Ability*				*T1*				*T2*			
Source	Df	Sum Sq	Mean Sq	F	Df	Sum Sq	Mean Sq	F	Df	Sum Sq	Mean Sq	F	Df	Sum Sq	Mean Sq	F
Pay T1	2	1.69	0.85	8.40***	2	0.97	0.48	4.74*	2	2.17	1.09	0.26	2	5.64	2.82	0.51
Pay Increase	2	0.15	0.08	0.76	2	0.25	0.12	1.22	2	5.22	2.61	0.64	2	9.84	4.92	0.89
Pay T1 x Pay Increase	4	0.25	0.06	0.62	4	0.46	0.11	1.11	4	20.69	5.17	1.26	4	11.31	2.83	0.51
Residuals	139	13.99	0.10		142	14.50	0.10		350	1434.62	4.10		142	786.25	5.54	

*p < .05.

**p < .01.

***p < .001.

**Fig 2:**
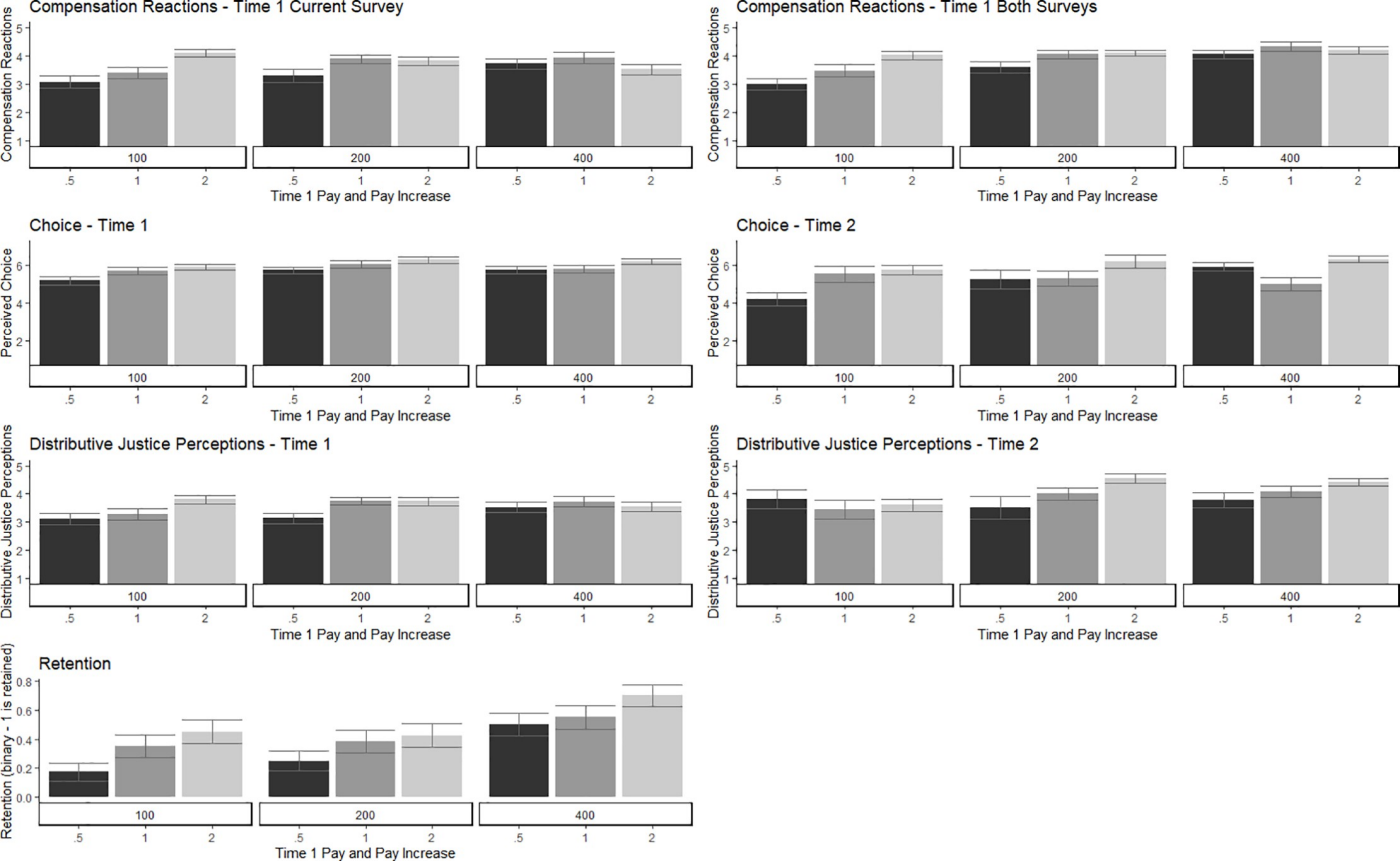
Significant effects for satisfaction and retention by experimental pay condition.

H2 predicted that pay would positively affect worker-participant satisfaction measured by post-test intrinsic motivation, compensation reactions, and distributive justice perceptions. Scores of each T1 satisfaction measure were regressed onto initial Pay, Pay Multiplier, and Total Pay (Tables 7 and 8, Fig 2). There were no significant effects of Pay on Enjoyment or Perceived Effort. There was a significant positive effect of T1 Pay and Pay Multiplier on Perceived Choice. There was also a significant effect of Total Pay on Compensation Reactions at T1. Participants initially receiving $0.50 with no increase (100% multiplier) in the second wave had the lowest compensation reactions, while any participant making a total of at least $3.00 generally scored highest. T1 Pay had a significant effect on T1 Distributive Justice with those initially receiving $2 scoring highest. A second regression was conducted for participants with scores on each T2 satisfaction measure (Tables 7 and 8, Fig 2). Again, there were no significant effects of pay on Enjoyment or Perceived Effort. Total Pay did, however, significantly predict Perceived Choice at T2. Of those receiving the largest increase in pay (400% multiplier), participants receiving an initial pay of $1 scored the lowest on Perceived Choice, but overall the lowest total pay of $1 resulted in the least perceived choice. Pay did not predict Compensation Reactions at T2. Total Pay positively affected Distributive Justice at T2. H2 was partially supported.

**Table 7:** Regression results for the effect of pay on worker-participant satisfaction and retention.

	IMI Enjoyment		IMI Effort		IMI Perceived Choice		Compensation Reactions (Current Wave)		Compensation Reactions (Both Waves)		Distributive Justice		Retention^a^
	T1	T2	T1	T2	T1	T2	T1	T2	T1	T2	T1	T2	
Coefficient	*B (SE)*	*B (SE)*	*B (SE)*	*B (SE)*	*Value (SE)*	*B (SE)*	*B (SE)*	*B (SE)*	*B (SE)*	*B (SE)*	*B (SE)*	*B (SE)*	*B (SE)*
Intercept	4.64 (.23)***	4.82 (.55)***	5.90 (.16)***	5.63 (.38)***	5.19 (.18)***	4.22 (.50)***	3.08 (.18)***	3.43 (.36)***	3.00 (.17)***	3.14 (.38)***	3.11 (.17)***	3.82 (.37)***	-1.55 (.42)***
Pay T1 ($1)^b^	-.14 (.32)	-0.54 (.67)	-.19 (.22)	.03 (.46)	.51 (.26)	1.32 (.61)*	.33 (.26)	-.07 (.44)	.48 (.24)*	.21 (.46)	.18 (.25)	-.38 (.45)	.93 (.53)
Pay T1 ($2)^b^	.28 (.32)	-0.05 (65)	.04 (.21)	.04 (.44)	.73 (.26)**	1.54 (.58)**	1.03 (.26)***	.35 (.43)	1.03 (.24)***	.58 (.44)	.70 (.25)**	-0.22 (0.43)	1.35 (.52)**
Pay Increase (200%)^c^	.39 (.32)	1.28 (.72)	-.16 (.22)	.19 (.49)	.56 (.26)*	1.03 (.65)	.23 (.26)	.47 (.47)	.60 (.24)*	.66 (.49)	.03 (.25)	-.30 (.48)	.45 (.55)
Pay Increase (400%)^c^	.45 (.32)	0.21 (.64)	.03 (.22)	.35 (.44)	.56 (.26)*	1.70 (.58)**	.65 (.26)*	.42 (.42)	1.05 (.24)***	.61 (.44)	.42 (.25)	-.05 (.43)	1.55 (.52)**
Pay T1 ($1) X Pay Increase (200%)	.16 (.46)	-0.01 (.90)	.32 (.31)	-.10 (.61)	-.20 (.37)	-1.25 (.81)	.27 (.37)	.37 (.59)	-.02 (.34)	.05 (.61)	.43 (.35)	.85 (.60)	-.30 (.72)
Pay T1 ($2) X Pay Increase (200%)	-.51 (.46)	-1.05 (.87)	.34 (.31)	.20 (.60)	-.19 (.37)	-.58 (.78)	-.5 (.37)	.22 (.58)	-.53 (.33)	.03 (.60)	-.09 (.35)	1.30 (.58)*	-.55 (.71)
Pay T1 ($1) X Pay Increase (400%)	.21 (.46)	1.05 (.81)	-.10 (.31)	-.30 (.55)	-.43 (.37)	-2.23 (.73)**	-.13 (.37)	.59 (.53)	-.20 (.33)	.35 (.55)	.02 (.35)	.70 (.54)	-.73 (.70)
Pay T1 ($2) X Pay Increase (400%)	-.80 (.46)	-0.27 (.78)	.17 (.31)	.34 (.53)	-.28 (.37)	-1.12 (.70)	-1.23 (.37)***	.55 (.51)	-.88 (.33)**	.35 (.53)	-.68 (.35)	.86 (.52)	-.50 (.70)
Model R^2^	.03	.10	.02*	.07	.07*	.16*	.07**	.19***	.13***	.18***	.05*	.14*	.069d

Note: SE = Standard error

^a^ results from logistic regression

^b^ Time 1 Pay treated as factor for this analysis; coefficients represent the effect in comparison to the referent group of (T1 pay = $.50).

^c^ Pay increase treated as factor for this analysis; coefficients represent the effect in comparison to the referent group of (T2 increase = 100%). ^d^ McFadden R^2^ reported.

*p < .05.

**p < .01.

***p < .001.

**Table 8:** Analysis of variance for effect of pay on worker satisfaction.

	**Intrinsic Motivation Inventory**																							
	*Enjoyment T1*				*Enjoyment T2*				*Effort T1*				*Effort T2*				*Perceived Choice T1*				*Perceived Choice T2*			
Source	Df	Sum Sq	Mean Sq	F	Df	Sum Sq	Mean Sq	F	Df	Sum Sq	Mean Sq	F	Df	Sum Sq	Mean Sq	F	Df	Sum Sq	Mean Sq	F	Df	Sum Sq	Mean Sq	F
Pay T1	2	1.89	0.94	0.45	2	6.65	3.32	1.57	2	2.55	1.27	1.30	2	4.20	2.10	2.11	2	19.84	9.92	7.32***	2	25.51	12.75	7.42***
Pay Increase	2	5.42	2.71	1.29	2	14.15	7.07	3.33*	2	0.26	0.13	0.13	2	3.91	1.95	1.97	2	12.22	6.11	4.50*	2	4.66	2.33	1.35
Pay T1 x Pay Increase	4	11.78	2.95	1.40	4	12.59	3.15	1.48	4	2.57	0.64	0.66	4	2.00	0.50	0.50	4	1.96	0.49	0.36	4	17.04	4.26	2.48*
Residuals	350	737.52	2.11		142	301.36	2.12		349	341.04	0.98		141	139.87	0.99		350	474.72	1.36		142	244.00	1.72	
	**Compensation Reactions**																				**Distributive Justice**			
	*Current Wave T1*				*Current Wave T2*				*Both Waves T1*				*Both Waves T2*				*T1*				*T2*			
Source	Df	Sum Sq	Mean Sq	F	Df	Sum Sq	Mean Sq	F	Df	Sum Sq	Mean Sq	F	Df	Sum Sq	Mean Sq	F	Df	Sum Sq	Mean Sq	F	Df	Sum Sq	Mean Sq	F
Pay T1	2	13.89	6.94	5.17**	2	9.34	4.67	5.08**	2	19.86	9.93	8.88***	2	11.26	5.63	5.67**	2	12.54	6.27	5.14**	2	6.59	3.29	3.51*
Pay Increase	2	2.58	1.29	0.96	2	19.32	9.66	10.52***	2	29.10	14.55	13.02***	2	19.70	9.85	9.92***	2	2.50	1.25	1.02	2	9.64	4.82	5.14**
Pay T1 x Pay Increase	4	19.71	4.93	3.67**	4	1.48	0.37	0.40	4	8.76	2.19	1.96	4	0.75	0.19	0.19	4	8.74	2.18	1.79	4	4.64	1.16	1.24
Residuals	350	470.46	1.34		142	130.47	0.92		350	391.12	1.12		142	140.96	0.99		350	427.17	1.22		142	133.21	0.94	

*p < .05.

**p < .01.

***p < .001.

H3 predicted a negative effect of pay on attrition. We used a logistic regression model, where the binary outcome of Completion was regressed on to pay (Tables 7 and 9, Fig 2). T1 Pay and Pay Multiplier significantly predicted whether a participant completed the second survey such that those with higher T1 Pay and a larger Pay Multiplier were more likely to complete the survey at T2. H3 was supported.

**Table 9:** Analysis of deviance for logistic model of effect of pay on retention.

	Retention				
	Residual Df	Residual Deviance	Df	Deviance	χ^2^ *p* value
Null	358	488.59			
Pay T1	356	476.85	2	11.74	0.003***
Pay Increase	354	456.43	2	20.42	0.000***
Pay T1 x Pay Increase	350	454.82	4	1.61	0.808

*p < .05.

**p < .01.

***p < .001.

## Discussion

The scientific community has expressed both excitement and skepticism about the value of MTurk Workers as a population. The purpose of this study was to explore whether pay was a motivator for Workers, specifically in a longitudinal study. Findings showed that pay mattered for satisfaction and attrition but not performance. The norms of MTurk exert significant pressure on Workers to do a “good job” regardless of their satisfaction, because they risk rejection of their work if their performance is not acceptable. Thus, if a Worker submits a task, it is probable that the task will be of high quality regardless of pay level or Worker satisfaction, even at very low pay rates. On the other hand, low pay will likely lead to a penalty to the Requester’s reputation. Regardless of acceptable data quality in an initial HIT, decreased satisfaction and increased attrition are likely to jeopardize future data collection efforts (especially for longitudinal studies) and undermine the success of the MTurk platform for researchers. Further, it is unethical. MTurk Workers view themselves as employees who are entitled to fair pay, generally US minimum wage. A Worker’s average compensation was only US$1.38 per hour in 2010 [43]. Little progress has been made here as recent research estimates the median hourly wage (taking into account the influence of unpaid work such as time spent searching for HITs or work on HITs that are ultimately rejected) is about US$2 per hour with only four percent earning more than US$7.25 per hour [44].

In the special case of multi-wave studies, the scope of the current study, it appears that worker-participants generally do not take the average of the two pay rates in determining fairness during the first wave. With the exception of initial participant satisfaction in the first wave and distributive justice at T2, there were few significant effects of the combination of pay increase and T1 pay on satisfaction, performance, retention or data quality. Rather, participant performance, satisfaction, and retention as well as the quality of their work all depend on the initial T1 pay. Lower initial pay generally resulted in worse outcomes. Participant satisfaction with compensation across both waves was dependent on the pay increase; participants were more satisfied with their compensation when their pay increase was steeper. Pay increase also affected retention and perceptions of justice in the second wave of the study.

There was mixed support that initial pay affects performance/data quality. Generally, data quality was not affected by pay. Personal reliability across personality measures did seem to increase as T1 pay increased, and maximum LongString was affected by total pay. However, data quality and performance did not seem to be affected by initial pay, pay increase, or total pay. Researchers’ concerns that MTurk Workers are only participating for the money may initially be warranted, but when considering longitudinal research other factors may be more important.

As expected, compensation reactions and distributive justice perceptions at T1 are typically related to T1 pay. Pay does not necessarily offer much intrinsic motivation, but participants do report more perceived choice as a function of pay. A decrease in perceived choice in T2 was possibly related to individuals with higher perceived choice in T1 exercising this choice and not returning for the second wave. Paired with the performance findings, this suggests that Workers are in a social context in which they have a certain level of choice over which HITs to accept (based on pay), but that after engaging in an unofficial contract with a Requester, their level of effort and the resulting performance do not change as a function of pay.

We took a similar approach to explaining our findings for the relationship between pay and satisfaction as Judge and colleagues [18] in their landmark meta-analysis of pay satisfaction. Helson’s [45] adaptation level theory suggests that individuals judge their current experiences based on a reference point that is adjusted as a function of previous experiences and contextual stimuli. As such, a pay increase may influence this reference point and lose its value over time. Similarly, Lucas et al. [46] discuss the effect of hedonic leveling whereby individual well-being stabilizes over time such that positive events affect those whose lives are already satisfying less than those with poor well-being. Based on this rationale, it would be expected that high pay would be most satisfying for individuals, like MTurk Workers, who have historically been underpaid for their work in addition to those who receive large pay increases over time after initial lower pay.

T1 pay and T2 pay multiplier significantly predicted retention, but there was not a significant interaction between the two. MTurk Workers may recognize T1 pay as an initial hurdle to participation, but after completing T1 tasks, they renegotiate their psychological contract about the value of participation relative to the time costs associated with returning for T2. Here, a higher pay increase represents a recognition from the Requester that the Workers’ time is valuable.

The current study makes a major contribution to current discussion surrounding ethical treatment of MTurk Workers by applying psychological principles of work motivation, psychological contracts, and pay. The findings are generally applicable to a new kind of virtual work environment similar to traditional labor halls of the industrial revolution. However, the inferences made based on these findings have three limitations which may offer guidance for future research in this area.

As a first limitation, by nature, the current study does not allow us to infer the psychological and motivational characteristics of those MTurk Workers who did not accept the HIT. Though non-respondents are admittedly a blind spot in any social science research, it is particularly important for this study because it indicates a possible preferred threshold for initial pay level. The MTurk platform allows Workers to sort and filter HITs based on pay, thus non-respondents for this study include those who never saw the HIT due to pay and those who outright chose not to complete it after previewing the task and comparing it with pay. The current study does not allow us to disentangle these two scenarios.

Secondly, age was not collected as a demographic variable. There is a lack of evidence to suggest that age is a significant determinant of motivation, especially in gig work [11]. Age and tenure are highly correlated and when controlling for the latter, age is typically not a determinant of pay fairness perceptions [47]. Given the fact that MTurk is an informal marketplace and does not represent a typical employee-organization relationship, the effect of tenure is unclear. However, meta-analytic findings from Bal and colleagues suggest age moderates the relationship between psychological contract breach and attitudinal outcomes, such that as age increased the negative relationship between contract breach and trust and organizational commitment weakened [48]. MTurk Workers are typically older and more age-diverse than other convenience samples used for social science research such as undergraduate students [1]. This makes age an interesting factor to explore in future research regarding variable expectations of the working environment and reactions to pay, justice perceptions, and psychological contract breaches.

Thirdly, the current study makes inferences about low pay on crowdsourced work platforms such as MTurk, but it does not consider the possible undue influence of excessive pay compared to similar tasks. The Belmont Report which is concerned with the fair treatment of human subjects states that “undue influence…occurs through an offer of an excessive, unwarranted, inappropriate or improper reward or other overture in order to obtain compliance” [7]. Though not particularly relevant for the tasks that participants completed in this study, other research which requires disclosure of socially unacceptable attitudes and behavior should be particularly concerned about undue influence especially with populations as vulnerable as underpaid MTurk Workers. Subsequently, future research should focus on the boundary conditions of an appropriate level of pay for social science research with a focus on finding a balance between exploitation and undue influence.

Despite differences between worker-participants and voluntary or student research participants, academic Requesters may not view themselves as employers. Nonetheless, they have an equal ethical obligation to all types of participants which should incorporate unique participant motivations and expectations and the psychological contract. We have shown that although the evidence does not suggest pay affects the overall performance of a Worker, it does affect satisfaction and attrition. We hope to demonstrate that MTurk can be viewed and understood as a unique workplace, with unique needs in terms of compensation, and Requester-Worker expectations. Attention toward these characteristics, as with any research participant population, is one of many critical determinants of retention in longitudinal research.
